# Parental strategies to promote theory of mind development in autistic children of color

**DOI:** 10.3389/fpsyg.2024.1347504

**Published:** 2024-04-18

**Authors:** Annahita Modirrousta, Yvette R. Harris

**Affiliations:** Department of Psychology, Miami University, Oxford, OH, United States

**Keywords:** autism, theory of mind, race, ethnicity, culture, parents, children, development

## Abstract

**Introduction:**

Autism Spectrum Disorder is characterized by an impaired ability to take other people’s perspectives, which is known as theory of mind. However, little is known about how theory of mind exhibits itself in autistic children of color and how parents foster their child’s developmental skills in communities of color.

**Methods:**

Two interviews were created to assess how parents appraise their child’s developmental skills and help their child grow: a perspective-teaching interview and a general developmental skills interview. Four families participated in the study; three children were African American or mixed, while one had an Asian mother. Parents were asked how their child exhibited various developmental skills and how they helped their child with those milestones.

**Results:**

Parents pointed at similar delays in theory of mind and other developmental skills, such as turn-taking and reading faces. They also provided similar strategies to teach those abilities, such as modeling and reinforcements. Several insights and common themes were found regarding autistic behaviors their children expressed and how those affected their parenting experiences.

**Discussion:**

These findings allow for a better understanding of parents’ experiences raising autistic children of color; future research could expand on their stories and create interventions tailored to these underrepresented communities.

## 1 Introduction

Autism spectrum disorder (ASD) is a neurodevelopmental disorder characterized by impaired social skills and repetitive patterns of behavior. The main two symptoms of ASD include “persistent deficits in social communication and social interaction across multiple contexts” and “restricted, repetitive patterns of behavior, interests, or activities” (American Psychiatric Association, 2013). However, research on autism does not fully represent certain demographics. For example, while White and male children have been researched extensively, minority groups such as children of color are hardly included or ignored altogether (Jones et al., 2020). As a result, autism research is unrepresentative of the population of autistic children^1^. The scientific literature is sparse with information regarding the experiences of autistic children of color and their families. This can prevent autism professionals from detecting symptoms in children of color and providing the correct diagnosis and treatments, which harms this vulnerable group.

In addition, certain aspects of autism have primarily been examined in White children but not in children of color (Jones et al., 2020). One of these is theory of mind (ToM), which is defined as the ability to attribute mental states to oneself and others and to understand that others have beliefs, desires, intentions, and perspectives that are different from one’s own (Berenguer et al., 2018). Earlier studies have shown a significant ToM impairment in autistic people; for example, Pastorino et al. (2021) found ToM impairments in children with ASD compared to their healthy peers and other patients with neurodevelopmental disorders. However, autistic children can still care for others’ emotions and thus develop empathy due to their ability to have empathic concern (Lockwood et al., 2013).

One of the factors behind this underrepresentation of autistic children of color is due to disparities in autism diagnosis and care resulting from structural racism (Broder-Fingert et al., 2020). For instance, racial and ethnic disparities have been found in autism-related services, with Black, Asian, Native American, and Pacific Islanders receiving fewer outpatient services compared to white children (Bilaver et al., 2021). Geographic access to autism resources is unevenly distributed across the United States, leading to autistic children of color having disproportionally lower access to services (Liu et al., 2023). Intersectional theory suggests that female and Black populations are diagnosed later than others because they do not fit stereotypical presentations of autism; as such, they face exclusion from research as well as tailored interventions designed for autistic children (Diemer et al., 2022). Finally, Black and Latino families have more challenges receiving high-quality health care, especially if their child has an ASD diagnosis compared against other developmental disabilities (Magaña et al., 2012).

Autism presents itself in culturally relevant ways, and parents react differently to those symptoms. For instance, in Asian families, there is a stigma against eye contact, which is considered rude (Tek and Landa, 2012). Because of this, Asian American children may not exhibit eye contact, a sign of proper development and a trait impaired in autism. Asian parents of autistic children also tend to exhibit specific patterns of behavior, such as redirecting energy towards the child, spiritual coping, and acceptance of disability (Luong et al., 2009). In addition, Dyches et al. (2004) found cultural factors that influence how African American parents raise autistic children, such as lack of access to healthcare, parental responsibility, and positive interpretation of disability. The U.S. Center for Disease Control and Prevention (CDC) reported that poverty and racial stigma contribute to delayed ASD diagnoses and less access to treatment for Black children compared to White children, despite similar ASD prevalence rates (Maenner et al., 2021). Finally, Tek and Landa (2012) found that Hispanic families with an autistic child appeal to authority, and strong family cohesion results in parents taking responsibility for their child’s disability. Mothers view their child’s disability as a gift from God and assume the role of a “sacrificing mother” to care for their child. The authors also found culturally different expectations of development, so milestones are expected at different times, and autism symptoms may not be detected as early.

Research has shown several characteristics of ToM development in different ethnicities. For instance, storytelling is a big part of African American culture, which teaches the ability to understand a character’s feelings and intentions, and false belief understanding was linked to African American children’s ability to understand and comprehend narratives (Mills and Fox, 2016). Also, ToM is exhibited differently in bilingual children than in monolingual American children (Liu et al., 2008). Kobayashi et al. (2007) found that different parts of the brain light up during ToM exercises between bilingual Asian Americans and monolingual American children. This suggests a potential difference in ToM between different ethnicities, whether it be neurology or cognition. Goetz (2003) also found that bilingual Mandarin-English children develop ToM faster and better than monolingual English or Mandarin children. This shows that in bilingual households where parents encourage their children to speak multiple languages, ToM is developed better. Being exposed to several languages helps children understand others better due to learning different ways of using language to relate to others. Since ToM plays a crucial role in social communication and executive functioning, early ToM capabilities can lead to later adaptive behaviors as well as academic success among autistic children (Memisevic et al., 2018; Roselló et al., 2022). However, in recent years, the understanding of ToM in autism literature has significantly changed. The idea that autistic individuals have a ToM impairment has been challenged, with a growing awareness of new theories explaining perspective-taking and empathy in autistic individuals. Many autistic scientists have been voicing significant criticisms of this hypothesis, which they claim reinforces negative stereotypes and discrimination against autistic people and excludes the autistic community from research concerning them (Holt et al., 2021). For instance, Milton (2012) addressed the growing awareness of a “double-empathy problem” in perspective-taking differences between neurodivergent and neurotypical people. Specifically, autistic individuals have difficulty taking the perspective of neurotypical individuals, but neurotypical individuals also have difficulty taking the perspective of autistic individuals due to a lack of insight into the minds and culture of neurodivergent people. However, the perspective-taking deficits disappear between autistic people because they have similar perceptions and social experiences and thus can understand each other more easily. This refutes the ToM deficit in autism theory by suggesting that perspective-taking struggles can go both ways between neurodivergent and neurotypical people. In the development of the current study, the experiences of autistic children of color were examined under the ToM deficit model. Nevertheless, it is important to stay updated on new theories and challenges to outdated ones.

The current study aims to examine ToM and developmental skills development in autistic children of color through parental experiences raising their children. The central research question guiding this research examines how parents help autistic children of color learn developmental skills, focusing on ToM. The specific questions that were answered are:

1. What perspective-taking strategies do parents teach autistic children of color to develop their ToM ability?
2. How do autistic children of color exhibit developmental skills and milestones?
3. How do parents teach autistic children of color how to learn these developmental skills?

## 2 Materials and methods

### 2.1 Participants

The participants in this study were four families with a child of color formally diagnosed with ASD, with the parents participating in the interviews. In this study, the children will be referred to as Child A through D. For children A through C, the mothers provided the interview answers; they will be referred to as Mom A through C. Mom A is African American, Mom B is Asian from Kazakhstan, and Mom C is mixed Black and White. For Child D, the father, who is African American, participated in the first interview, while the mother, who is White, participated in the second interview. They will be referred to as Dad D and Mom D. The age range of the children was 4 to 5 years old. All children in the study were verbal except for Child A, who was nonverbal. The demographic information for the families can be found in Supplementary Table S1, made in reference to the demographics table in Habib et al. (2017).

Several recruitment tools were used to reach out to parents. Recruitment emails were sent to autism centers and schools across Ohio that cater to preschool children as well as national autism organizations. Facebook autism groups with a focus on self-advocates were contacted through Messenger with information about the study. Application forms were submitted to major autism organizations to advertise to parents and were posted on their websites. All recruitment messages described the purpose of the study, details about the procedures, and inclusion/exclusion criteria while emphasizing advocacy and working with the community. The study’s approval form from the Institutional Review Board (IRB) was provided along with a recruitment flyer, which included a description of the study, participation criteria, and contact information. These criteria included Asian American, African American, or Latino/a children between the ages of 4 to 7 with an ASD diagnosis and no comorbid developmental disorder. The study also required a good internet connection and access to Google Meet, the software used for the virtual interviews. Initially, the study was designed to be in-person; however, due to the ongoing coronavirus pandemic during the recruitment process (taking place in 2020–2022), the study shifted to a video interview. This allowed for expanding recruitment across the country rather than solely focusing on the region.

The children in the study had an official diagnosis of ASD, and parents provided documentation of their child’s evaluation and diagnosis. Parents did two rounds of interviews and received an honorarium of a $30 Amazon gift card upon completion of the second interview. Mom A only completed the first interview and did not provide answers for the second interview questions.

### 2.2 Measures

The initial methodology of the study was a mixed-methods study and included additional measures for the children. The first three interviews with Parents A, C, and D involved two ToM tasks for the children, the Sally-Anne False Belief Task (Baron-Cohen et al., 1985) and the Unexpected Contents False Belief Task (Hogrefe et al., 1986). These tasks would have been analyzed quantitatively to determine how perspective-taking teaching strategies predicted ToM ability. However, due to the small sample size, the methodology was changed to focus on qualitative analysis of parents’ interviews and an emphasis on their experiences with raising their children and helping them with developmental skills. In addition, the original methodology only included the first interview, but the second set of questions regarding broader developmental skills was added as an additional interview after submitting a study modification to the IRB and getting approval. This allowed parents to elaborate further on their experiences and have a fuller image of their strategies to foster their children’s development.

#### 2.2.1 Demographics questionnaire

A demographics questionnaire was given orally to the parents to obtain information about themselves and their children. The results were used to screen participants’ demographic information, including education level, employment, socioeconomic status, and ethnicity.

#### 2.2.2 Interview on parental teaching of perspective-taking (PTPT)

A researcher-created interview was given orally to assess how parents teach perspective-taking. The questions were based on the literature regarding different strategies that can contribute to ToM development. Parents were asked questions about storytelling (Mills and Fox, 2016), mimicking (Tunçgenç et al., 2023), social activity (Berenguer et al., 2018), language development (Goetz, 2003), and environmental influence (Smogorzewska et al., 2020). Each topic contained several elaborative questions regarding how those strategies are used to foster their child’s ToM ability. Example questions from the PTPT include: “How often do you read stories to your child?,” “When reading stories, how often do you teach your child how to understand the characters’ thoughts and feelings?,” “How often do you teach your child how to take the perspective of another person by showing and mimicking?,” and “How often do you spend nurturing your child?”

#### 2.2.3 Theory of Mind Inventory

The Theory of Mind Inventory (Hutchins et al., 2012) is a 42-item ToM test that was given orally to the parents to gauge their child’s ToM based on how they see it. Each of the items on the ToMI is a specific dimension of ToM. This test includes three subscales (Early, Basic, Advanced). The “early” subscale refers to ToM development during late infancy and early childhood, the “basic” subscale refers to ToM development during the preschool years, and the “advanced” subscale refers to ToM abilities emerging during late childhood and continuing into adolescence. Examples of early ToM development include the ability to show things and pay attention when people point to something. Basic ToM development includes the ability to understand words like “think,” “know,” and “if.” Advanced ToM development includes the ability to understand plays on words and sarcasm, but considering the participants were 4 to 5 years old, the Advanced subscale of the ToMI was excluded.

The scores were calculated by assigning values of 1 to 5 for how much parents agreed with each statement (1 for definitely not, 2 for probably not, 3 for undecided, 4 for probably, 5 for definitely) and summing up values for the 26 questions to create an overall score. Although the original test only included a Likert-scale quantitative measure, the study also transcribed parents’ verbal answers for qualitative analysis as part of the oral interview.

#### 2.2.4 Interview on parental teaching of general developmental skills (PTGDS)

A second researcher-created interview was given orally to assess autistic children of color’s developmental skills and how they experience developmental milestones. The interview included additional questions regarding how parents helped their children develop these skills, which were taken from the CDC’s list of developmental delays in autism (Centers for Disease Control and Prevention, 2022). Parents were asked questions regarding their children’s ability to use skills such as turn-taking, playing pretend, sharing interests, eye contact, physical gestures, reading faces, and looking when someone points to something. For each topic, parents were asked how they encouraged and helped their children learn developmental skills and offered several strategies to improve those skills. Those strategies included modeling, reinforcements, gestures, and playing games. Example questions from the PTGDS include: “How good is your child at turn-taking?,” “How do you help your child learn how to play pretend?,” “How do you encourage your child to share interests?,” and “How good is your child at reading facial expressions and determining someone’s feelings?”

### 2.3 Procedure

Parents interested in participating in the study contacted the researcher using the email provided in the recruitment materials. They were thanked for their interest in the research and sent an email with a Qualtrics survey containing a consent form. The written research consent explained the purpose of the study, research and societal benefits of the study, a detailed procedure of the study with the duration, benefits and risks of participating, a disclaimer of the voluntary and confidential nature of the study, and contact information of the researchers. Parents would agree to the consent and fill out their name and date. The form included additional consent for recording interviews, which explained that the interviews would be recorded and transcribed for use in presentations and papers without personal information attached. Parents would then consent to have their interview recorded and could choose whether they wanted their voices and pictures used in the dissemination of the study. After completing the survey, parents were thanked and asked to provide the researcher with a scan of their child’s official diagnosis and their availability for Google Meet.

The researcher then contacted parents to schedule an interview on Google Meet, which lasted approximately 30 min. The interviews took place using a webcam and WeVideo for the screen and audio recordings. During the first interview, the researcher read an oral summary of the consent form, and parents verbally accepted the consent. Then, parents answered the questions on the demographics questionnaire, the PTPT, and the ToMI. Parents were told to answer the PTPT and ToMI to the best of their ability and talk in as much detail as possible. When appropriate, the researcher asked parents to elaborate on their answers. The researcher also asked parents at the end whether they could think of other methods they used to teach perspective-taking and asked them to elaborate on those strategies. The researcher then thanked the parents for participating at the end of the interview.

Months after the first interview, the same parents were contacted for a second interview. Parents were told about the nature of the new interview and that they would be rewarded with a $30 Amazon gift card for their participation. Once the interview was scheduled, the researcher met the parents on Google Meet and explained the additional interview questions on the PTGDS. Parents were instructed to elaborate on answers and were told they could skip any questions they felt uncomfortable answering. After answering the questions, parents were thanked for their participation and for sharing their stories. Finally, they offered their information so that the gift card could be mailed to them.

### 2.4 Data analysis

The Google Meet recordings were uploaded into a Google Drive folder, which the researcher only shared with the graduate advisor and undergraduates in the Harris Parent–Child Interaction Lab at Miami University. The undergraduate students manually transcribed both interviews for Parents C and D, and the interviews for Parents A and B were transcribed into text documents using the Happy Scribe software. The machine-generated transcriptions were proofread and fixed manually, and all transcriptions were saved in Google Docs in the Drive folder. The demographic information for each parent–child dyad was filled into the demographics form and kept in the folder.

The interviews were analyzed qualitatively by the primary researcher using thematic analysis (Braun and Clarke, 2006). Concepts of ToM and developmental skills development were taken from the literature to create the interview questions; then, similarities and themes were extracted from the parents’ answers. The researcher read over the transcripts several times to become familiarized with the parents’ answers, wrote down a summary of each answer, and then separated the responses into categories based on similar patterns to create the themes.

The results were separated into three sections: *perspective-taking strategies, general developmental skill strategies,* and *recurring themes* found to be common in the different parental experiences. For the *perspective-taking strategies* section, parents’ answers to the PTPT interview were examined while similarities and differences in parental strategies were drawn and explored; this would address the first part of the research question. For the *general developmental skill strategies* section, the emphasis was on similarities and differences in parents’ strategies to teach their children developmental skills; this would address the second and third parts of the research question. For the *recurring themes* section, general recurring themes spanning across interviews were established. In each section, examples from the four parents’ interviews will be provided and compared.

## 3 Results

### 3.1 Perspective-taking strategies

The first interview addressed the first research question, namely, what perspective-taking strategies do parents teach autistic children of color to develop their ToM ability? Several elements of perspective-taking were analyzed; a summary of the themes described by parents will be provided, along with examples from each parent. For the first interview, Dad D provided the answers for Child D.

#### 3.1.1 Storytelling

The parents would read story books and nighttime stories to their child, with Mom A mentioning she also let her child watch storytelling videos on YouTube. Some parents also used social stories, which are stories designed to teach autistic children how to grasp social concepts and learn appropriate social skills on how to interact with others. Mom B explained:

I’m mostly focused on his interest because that’s like what is there. So depending on his interest, I also try to bring some interest in the season. For example, now it’s Christmas. I’ll get a couple of Christmas books and some Christmas books with some characters and something else. Usually, I also have social stories… like words are not for hurting, do not hit.

When telling stories, the parents would act out the characters’ feelings to teach their child how to understand their perspectives. They would describe how characters felt or make facial expressions to mimic the characters. However, some children struggled with paying attention or sitting still during story time, which made storytelling more difficult for parents. Dad D explained:

I’ll just act it out. Like if he’s sad, I’ll show “sad” (sad expression) or if the character is happy then “happy” (cheerful expression).

#### 3.1.2 Mimicking

The parents would teach mimicking through stories; Mom B mentioned making different voices for characters and asking her child what he thinks the characters are thinking and feeling and what their intentions are. They would also act out emotions and facial expressions to teach their child how to behave in public. Mom A explained:

That’s basically like an everyday or every other day thing, especially with the sisters. They’re all like a year or less in part. So sometimes they’ll make me cry. Sometimes they’ll make each other cry. And I’m like, look, do this. See, she’s crying. See? She’s hurt. See, she’s upset. Look, I’m hurt. Look, you are hurt. So that’s like, every other day.

Some parents mentioned using verbal and physical praise when their child recognizes faces and responds with the proper emotion. Mom A would give her son high fives, grab his hands, or say, “good job.” However, Mom B said she does not like using reinforcements. She explained:

No, I do not like reinforcing his stuff because I understand that cognitive empathy he needs to process, which he’s building. He just has to get his own understanding of motivation of this person, and he’s still kind of fighting between what he sees and what he wants to see.

#### 3.1.3 Social activity

The parents mentioned their child would prefer playing alone in daycare or class rather than participating in social activities. The children would sometimes engage in play sessions depending on who was around and where they were. However, they would mostly spend time by themselves and only engage with close friends or acquaintances. There was the additional constraint caused by the pandemic, which Mom B mentioned as a factor that made it harder to go outside and interact with others. Mom A explained:

It depends on who it is and where we are. It’s very rare… I would say, like, maybe it’s probably like a good 25 to 30% of the time he’ll actually engage and be aware. Other than that, it’s by himself or like in his head. If you saw him in the car, it’ll be like that.

Some parents would use modeling and verbal cues to encourage their child to engage socially. For example, Mom B explained how she teaches her child how to speak to people and ask questions, such as when ordering food at a restaurant:

On Sunday, he went to the restaurant and the waitress is working. And he’s like, “hey hey hey!” (bangs fists on table) And I said “no, you do not speak in heys.” So he’s learning how to speak. It’s tricky.

#### 3.1.4 Languages

All the children were monolingual, but Child B used to speak Russian and has now forgotten most Russian words except swear words. Child A is nonverbal and cannot verbalize English but somewhat understands it when spoken to him. The other children were proficient in English and could speak full sentences using pronouns and prepositions. However, parents also pointed at language delays such as articulation and not expanding on sentences. Parents spoke highly of their child’s language ability, such as Mom C:

He’s pretty proficient, yeah. He understands a lot; he is very well versed. It’s amazing. He wasn’t talking about a year ago, and now he will not stop.

#### 3.1.5 Environment

All parents emphasized building a very supportive and positive environment for their child. Moms B and C, who are stay-at-home moms, stated their job was to nurture and care for their child. Mom B explained:

I think I spend most of the time just nurturing him, hugging and kissing because it’s part of my culture, it’s part of the expectation. When he’s not feeling well and grumpy. So he does not allow me to do these things, but yeah, he’s my baby.

Parents stressed their efforts to create a comfortable home and help their child as much as possible. Their parenting style was focused on warmth. Dad D explained:

I grew up being tough, but then I have to realize that I cannot be tough with him. So I have to, you know… just remember I gotta be softer with him.

#### 3.1.6 ToMI

The parents’ ToMI scores are listed in Supplementary Table S1. Total scores were calculated by adding the scores from each question, with a total possible score of 130. Parents mainly provided answers to the ToMI using only the wording from the rating scale (“definitely not” to “definitely”) with short comments as explanations. However, Mom B, who had the second highest ToMI score of 93, gave a lot of elaboration on her answers to the ToMI. She was also very optimistic about her son’s abilities and spoke passionately throughout the interviews. Mom C, with the highest score of 96, also provided enthusiastic and positive responses to the interview questions. Her tone during the interviews was lively, and she offered a lot of praise for her son. On the other hand, Dad D had the lowest ToMI score of 59 due to answering “definitely not” on 15 of the 26 questions. Parents D also gave shorter answers to the interview questions and were less optimistic in their tone and responses.

### 3.2 General developmental skill strategies

The second interview addressed the second and third research questions. Regarding how autistic children of color exhibit developmental skills and milestones, parents explained how their child exhibits those skills; as for how parents taught those children how to learn these developmental skills, parents elaborated on how they encouraged the development of those skills. Mom D provided the answers for the second interview instead of Dad D. Mom A did not respond for the second interview, so only Moms B, C, and D provided answers to the PTGDS questions.

#### 3.2.1 Turn-taking

The parents mentioned their child struggling with taking turns. Some children would not realize that it was not always their turn. The children would play video games and board games such as Candy Land or Sorry, allowing parents to encourage turn-taking. However, some children found it challenging to sit still and pay attention. Mom C explained:

We cannot make it through the whole game because of his inability to focus and pay attention that long, and also him wanting to have his turn, which usually ends into some kind of fit. But we try.

Parents would teach turn-taking through verbal cues such as “my turn, your turn,” as well as modeling to guide children through the process. Some parents would also use books and social stories that show characters taking turns. For instance, the “nice hands” social story teaches children how to use their hands during playtime. Parents would also use cards and pictures to show game instructions and reinforce those skills. Mom C explained:

At first, he struggled a lot with that. It was very hard for him to understand that it was not always his turn. He’ll always want to go twice or skip it. So we had to work a lot with the reverse “my turn, your turn” and sitting him down and really guiding him through that process. Every now and then he gets antsy with not wanting to wait for his turn and wanting to go again. There is a game that’s like “skip a turn.” That’s very difficult for him to lose his turn, cause that’s what the card says. He does not quite think that’s fair, even though it’s part of the game. So really just explaining it to him, saying “that’s what the card says and you have to follow the directions.” It has been pretty much just a really long process, very repetitive.

#### 3.2.2 Playing pretend

Children B and C loved to play pretend. For instance, Child B played pretend with his sister and enjoyed creating characters to play, such as doctors and kings. Child C enjoyed playing pretend by creating figurines from Play-Doh or pretending to drive a car. Moms B and C also praised their child’s imagination and creativity. Mom B explained:

Oh, he’s amazing. He loves it. So (child name) loves animals. So he’s playing in the zoo and I have like messages from his teacher. She showed me… she said, “your son has such a great day.” So he was playing a doctor. He loves it… Last time he was on the stage so it was a museum… They had a stage that was set up and played that he was a king. He played with his sister like a monster. But this is more like hustling wrestling game and tried to impersonate some other stuff. We do a lot with pretend game. We do a lot.

The parents mentioned modeling as a way to encourage pretend playing. Parents would enact imaginary scenarios and tell their child to look at them and imitate their behavior. Mom B also mentioned using her child’s special interest, such as monkey figurines, to create pretend games.

On the other hand, Mom D said her child is not good at playing pretend and playing with toys creatively. She explained:

So his older brother kind of models it for him and tells him like, “we are going to play house, you are gonna be the dad and I’m gonna be the child,” but it just… it’s very confusing and difficult for him… when he starts playing with him, he just ends up lining all of the toys up instead of playing.

#### 3.2.3 Sharing interests

The parents mentioned their child would share interests, but it usually consisted of their special interest, which they would discuss for long amounts of time. They would get excited about their special interest and share it with as many people as possible, but it would make up most of their interest sharing, and they would not often share other topics. The children would also be indifferent to others’ interests. Mom C explained:

It goes in one ear and out the other, pretty much. He does not grasp it. I’ll say “I like purple” and he’ll go “I like all the colors.” It’s him and him.

Although the children did not need encouragement to share their special interests, some parents reinforced their child’s sharing through prizes and encouragement. Mom C explained:

It’s a lot of reinforcement… “I like how she told me this story, I love and get really excited, I love that she built this character”… and just a lot of boosting him up, patting him on the back. A lot that gets him going.

#### 3.2.4 Eye contact

Moms C and D said they encouraged their child to develop eye contact by pointing and gesturing at the eyes. They would work with special education teachers in therapy to teach eye contact to their child. Parents were instructed to use verbal cues such as “look for the face” and “talk to the face.” Child C always had good eye contact and never struggled, whereas Child D would only make eye contact on his terms and look down when others tried to talk to him. Mom D explained:

He will on his own terms, but if you start looking at him or trying to address something with him, then he instantly starts looking down… with his ESE teacher, she has us doing it too. She will tell him (child name), then will get on his level, “look us in the eye when you are talking to us.” And that’s what we do at home too.

On the other hand, Mom B said she would not force her child to make eye contact. She explained:

You know, it’s a tricky part… because as me, as a Kazakh, we do not look to some stranger’s eyes compared with Western folks. So it actually was never, like, so much like… even this measurement and eye contact. When we do the same screening and everything else. I was like, no, he just kind of pays his attention. I mean, I do these things. So he has an eye contact, and I never was, like, forcing him to have eye contact because I know it’s difficult.

#### 3.2.5 Physical gestures

The parents mentioned their child’s delay in learning to use physical gestures. Some children would not point or use gestures, although Child D points a lot when he wants something. Parents would encourage gestures through modeling and guiding their child’s hands with their own. Mom B mentioned using toys and musical games such as “if you are happy and you know it, clap your hands” to teach hand clapping. Mom C said she tried teaching her child sign language, but he could not grasp it, and she has not modeled the behavior since. Meanwhile, Mom D would not model gestures for her child. She explained:

I actually encourage him to use his words. I’ll literally tell him, “use your words if you want something…”. because he is high-functioning, so we are trying to encourage him to use words instead of gestures. So we do not model any of that with him.

#### 3.2.6 Face reading

The parents mentioned their child struggling with recognizing facial expressions and feelings. For instance, they would not be able to pick up if someone is upset and detect the emotion in the face. Mom B said it was especially hard with more complex feelings and mixed emotions. She explained:

He is good, like six basic emotions. He can read it. When it’s something kind of mixed… the tricky part with (child name), when you have two emotions, he will recognize one of them. You can be sad and angry at the same time, you know… he will read one of the emotions. He will recognize only one basic, depending on his own mood and how much he feels like secure at this time. So that’s kind of funny. And like explaining to him the layers and complications that the emotions are different, it’s so difficult… you can have a mixed feeling and it’s not one feeling. Basically, kids have learned that it’s one feeling. Yeah. But actually, it’s not one feeling. It’s never one feeling, it’s a complex feeling.

Parents would teach their child this skill by showing pictures of faces and making them recognize the expression. The children would have to identify the feeling for each face and name the emotions shown. Some parents would also model the behavior by pointing to an angry face and encouraging their child to make the same face. They named books, toys, and television as tools they use to teach face reading. Mom D explained:

Sometimes… with sheets, teaching him feelings words that has like colors with faces on them. So that way he kind of associate colors with different faces. The sheets are like a picture. So it’s on the fridge at home and that’s what we are trying to get him to learn.

#### 3.2.7 Looking at pointing

The parents mentioned delays in their child’s ability to look when someone is pointing. For instance, Child B would prefer to pay attention to things on his own accord rather than focusing on where others were pointing. Meanwhile, Child C could not initially focus on the finger but eventually learned the skill. Mom C explained:

It took a while, I’m trying to think back to when that might… probably at two and a half, almost three he started doing it, because at first he would not. Like look over there… (points). He’d just be oblivious (looks around with head) and look at different directions. But closer to three I think he finally clued on to “hey, that’s what they want me to go” and something like that.

Parents encouraged this skill by modeling the pointing and giving verbal cues. For example, Mom B would tell her child “look there, look there,” while pointing at things to direct his attention. Mom C explained her modeling:

Yeah, a lot of modeling it… look over here and dad would run over and show him what I’m pointing at. Come backing it out. Reinforcing him when he did… encouraging him when he did do what we want him to. Different rewards.

#### 3.2.8 Other developmental delays

The parents mentioned similar developmental delays with motor skills due to weaker muscle tone. Children showed impairments in fine motor skills, such as holding a pen and using utensils. They also struggled with gross motor skills, such as kicking a soccer ball and riding a bicycle. Parents similarly listed speech delays with articulation, sounds, and word construction. The children were also delayed in writing, having difficulty recognizing letters and writing sentences.

Parents would use modeling to guide their child’s movements. For instance, Mom B would teach her child how to ride a bicycle and catch and throw balls. Similarly, Mom C has been trying to teach her child to kick the ball straight and not turn his foot outwards while playing soccer. She would also use her hands to help him properly use spoons to feed himself. She explained:

He had a hard time with feeding (spoon to mouth movement) and using his wrist. So we had to do a lot of modeling, hand over hand, to get him to… even now he does not realize his wrist can go like that (shows wrist turning). So he’ll do stiff arms and he’ll use the elbow (whole arm goes up while eating with spoon). So that’s something we are still working on.

### 3.3 Recurring themes

Based on Braun and Clarke (2006) process of thematic analysis, several themes were drawn out from the interviews outside of the topics from the structured interviews.

#### 3.3.1 Special interests

Most of the children in the study experienced a highly focused interest in specific narrow topics called special interests, which parents used to teach their children developmental skills. The parents mentioned their child being excited about their special interest and engaging in infodumping, which is the tendency for autistic people to “dump” as much info as they can about their special interest and go on about a specific topic. The children would sometimes ignore other people and turn the conversation into the topic of their special interest. Mom C mentioned her child’s special interests being Plants vs. Zombies and Play-Doh. Mom D explained that her child gets very excited about his special interests, Fortnite and baby Yoda, and would talk nonstop about them.

Parents would also use their child’s special interest to teach skills, such as telling stories and playing with figurines associated with the special interest. For instance, Mom B mentioned she has her own special interest, which is books, that she uses when teaching her child. She explained:

He has some basic interest and usually I try to use his (special) interest for something that he does not have a lot of skill, like fine motor skills. And then I take the figures which he likes and play some different games. We used to play a lot with like five little monkeys. We had this, I have this monkey and he was like pretending, and then he started pretending that he wanted to be the monkey, like “five little monkeys jumping on the bed, one fell off and bumped his head.” Then he would start pretending that he bumped his head. And he’d start playing and he’d become himself the monkey.

#### 3.3.2 Social stories

Parents mentioned using social stories to encourage their child’s development. These stories would help autistic children learn how to discern socially appropriate and socially inappropriate behavior. For example, Mom B explained in the first interview that she uses social story books to teach her son to be kind by not using hurtful words and not using his hands to hit others. Mom D mentioned using the “nice hands” social story to teach her child to use his hands for nice things; Dad D expanded on that and explained that the social stories would be coupled with visual boards to show their child how to use his hands visually. Mom C explained:

I use social stories both in his OT and in his speech therapy. We use those a lot with him and showing him in the story that it’s Suzy’s turn or your turn and who’s next. And letting him see how that played out in the social story before trying to do it. It’s definitely helpful.

Giving him a pencil or a crayon was really hard. He’s just not at the point where he’ll write… a sentence, a short sentence, and that’s taken… all of school since August, to get him on that level. He does prefer to use, like, markers instead of crayons or pencils. His site says there were some… I guess his body picks up on like the crayon breaking down or the lead coming off the paper, and that sound it makes. He’s very in tune to that and that might be the disruption there, the texture. So that’s been challenging.

#### 3.3.3 Applying concepts

Some of the children had a difficult time applying concepts from games or stories to real life and differentiating between fiction and reality. Mom C mentioned her child could identify facial expressions and feelings in social stories, books, and TV, but he could not apply that skill to real-life situations. She explained:

He can identify it in a social story or a book or on TV. He just does not seem to apply that in real life situations. Like somebody who’s upset. That just does not connect for him, he does not pick it up as often… There’s just a disconnect in the translation. If someone is upset, they are not happy. When you are playing with them, stop doing what you are doing. He understands it textbook-wise, but not real life wise.

In addition, Mom D stated that play-pretend games are confusing for her child. She explained:

It’s very confusing and difficult for him. When he goes and tells other people, it’s as if it happened and not that it was playing… The only pretend play he does is the house playing. Other than that, he does not do pretend play. Cause he’ll tell you that… it’s not real.

#### 3.3.4 Focus on strengths

Parents similarly emphasized their child’s strengths whenever they could, specifically their imagination and unique insights. Dad D said his son speaks “very well for his age” and Parents D emphasized being gentle and supportive. Mom B said her child is “amazing” at playing pretend and creates countless imaginative scenarios in his games. She also stated she is proud of her son’s language skills after only 2 years of speaking and believes in finding strengths in her child’s development. She brought up her child’s caring and empathetic nature, such as wanting to protect his mother and seeing villains as good guys in stories. She explained her child’s interesting insights:

I think it’s just so enjoyable for me, that when he comes and tells me something… sometimes it could be too much, but it’s something that I really enjoy for him to come in and tell me something is going on… So he was like, “oh, mommy, you know this? Mommy, do you know this? Oh, mommy. I’m gonna do this.” I just was like, “wow, you are telling me something cool.” And he has some interesting insight. He comes to me and I’m like, “who’s your superhero?” And he’s like, “Mommy, you are my superhero.” I’m like, “wow, that’s cool.” I’m like, “why am I your superhero?”

Mom C echoed the sentiment and said her child’s English proficiency is “amazing,” and he would talk nonstop nowadays after not being able to speak the year before. She also praised her child’s imagination and keen sense of creating associations based on vivid prior memories. She explained:

Oh, he’s incredible. His imagination is so far beyond and some of the stuff he comes up with is just out of the weirdest places from prior memories… like weeks ago he’ll just randomly remember something and be pretending it. That was a newer skill… well I want to say not until he was like three and a half or four-ish, he could not really play imaginary play. It took him a while, because even playing kitchen was hard for him to repeat that. But now that he is five, he does have an imagination and it’s quite active.

#### 3.3.5 Parental responsibility

The parents expressed their responsibility in raising their child. One of those responsibilities was to provide nurture and care. Dad D emphasized being soft and affectionate with his child and took responsibility for not being harsh like how he grew up. Moms B and C, being stay-at-home moms, spent most of their time nurturing and caring for their child. Mom C answered:

All day long, (laughs), that is my job. All day long. He gets all my attention… sole attention… at least 6 hours a day.

Another parental responsibility was creating a supportive environment for their child, which was addressed in the Environment subsection of the Perspective-Taking Strategies results. To elaborate, Mom B stated that she coaches parents of autistic children to focus on supporting and appreciating their child’s neurodivergence and development. She also emphasized building a circle of neurodiverse people around her child. She explained:

You know, neurodiverse people tend to choose neurodiverse people. So I need to make a circle of neurodiverse people around him by accepting him and he feels comfortable enough in order for him to thrive. And then I can bring him to neurotypical people.

#### 3.3.6 Parental strategies

The parents mentioned several similar strategies to help their child’s development, although there were some distinctions between parents.

##### 3.3.6.1 Modeling

Parents listed modeling, or imitating a particular behavior, as a way to teach developmental skills. For instance, Mom B would model physical gestures by guiding her child’s movements with her hands. Mom D said her child’s older brother would model pretend playing for him by playing the role of the child and teaching Child D how to play the role of the dad. Mom C explained her use of modeling:

We show him a lot of modeling, a lot of “hey, do what mommy is doing.” We did that a lot, follow dad, you are out. So imitating him.

##### 3.3.6.2 Verbal directions

Parents would provide verbal cues in addition to modeling to encourage the desired behavior. When playing games, parents would verbally announce whose turn it was and who would play which role. They would also explain what others are thinking or feeling and give directions verbally, such as “look over there” and “look us in the eye.”

For example, Mom A would describe the actions of people and animals around them. She explained:

We go on walks a lot, and during the walk, I’ll be like, “look, look at the animals together, like together…”. or something like that. It’s a lot of animals. Definitely. Like outside activities during, like, a movie. If something bad happens… I’ll be like “oh my gosh, oh my goodness… look at what they did, they are running, they are in trouble. We got to go. Oh yeah.” Or if it’s like sad, it’s like “aw, they are so sad. Look at them.”

##### 3.3.6.3 Reinforcements

Most parents mentioned reinforcements as a method to encourage their child’s desired behavior. For example, Mom A stated that she reinforced her child’s behavior through prizes and physical gestures such as high fives. Some parents would also praise their child when engaging in the desired behavior. Mom C explained:

This is what we are doing, we are practicing speech… “Okay, good job! All right, now you can go play… all right, come back and let us do it again.”

On the other hand, Mom B said she does not like to reinforce her son’s behavior. She wants Child B to learn on his own and get his own understanding of cognitive empathy and people’s motivations. She explained:

I never use any rewards (for him to) come and speak with me. It just was like, my and his relationship, which actually builds the skill.

#### 3.3.7 Mentions of culture

The study was designed to find cultural differences in parental experiences and strategies to teach developmental skills in families of color. However, only Mom B mentioned how her culture shaped her parenting. She brought up her Kazakh culture several times throughout the interview and explained how it played a role in her experiences raising her child. For instance, she brought up the differences between Kazakh and Western parents regarding teaching eye contact and nurturing her child. She also mentioned the cultural differences in gestures:

I guess it’s also the cultural aspect because my gestures from my country and the way how I express myself in nonverbal languages… it’s absolutely different from how most of the American folks do it.

Mom B also emphasized her nationality by referring to her Kazakh culture rather than broadly mentioning Asia. She raised her child in Kazakhstan when he was a toddler and later moved to the United States, meaning that, unlike the other parents, she was a first-generation immigrant. She was also the only parent who responded to the interviewer mentioning culture and shared her experiences with racism and xenophobia. In contrast, the other parents did not respond to the interviewer’s mentions of race and ethnicity.

## 4 Discussion

This research aimed to examine the different strategies parents used to encourage ToM development and other developmental skills in autistic children of color. The specific areas of interest were how children exhibited certain developmental skills such as ToM and how parents taught perspective-taking and other skills to help their children develop. Parents pointed at similar areas of developmental delays, such as turn-taking, reading faces, and language (both verbal and written). They also cited similar strategies to encourage their children’s development, such as modeling the desired behavior and using verbal cues.

### 4.1 Common experiences

Several common themes were found among the four families regarding their experiences raising their autistic child and teaching perspective-taking. For instance, all parents named storytelling a common activity in which they engaged with their child. Mom A mentioned YouTube story videos, while the other parents mentioned reading books and social stories. Consistent with the findings of Mills and Fox (2016), parents would act out the characters’ feelings and explain what the characters felt or thought, which encouraged their children to understand and comprehend the story narratives.

Parents also emphasized their child’s developmental strengths, using positive words such as “amazing,” “incredible,” and “interesting” to refer to their child’s imagination and creativity. They also praised their child’s progress and were impressed by how quickly they picked up developmental skills. Mom B’s optimistic mindset about her son’s autism was consistent with the findings of Luong et al. (2009), which found that Asian parents who have come to terms with their child’s autism do not see the condition as something to “fix,” but rather something to nurture and help their child grow. Likewise, the other parents’ experiences were consistent with Dyches et al.’s (2004) findings, which mentioned that African American families may interpret autism and disabilities positively.

Another similarity was the feeling of responsibility in taking care of their children. Mom B emphasized nurture as part of her job as a stay-at-home mom. This mirrors findings by Luong et al. (2009), stating that Asian parents of autistic children may become very involved in their child’s development and focus their energy on helping their child, many choosing to become stay-at-home parents to invest in their child’s growth. Similarly, the other parents mentioned they tried to make their child’s environment as nurturing and supportive as possible. This supported the findings of Dyches et al. (2004), which suggested that caring for children with disabilities is a familial responsibility in African American households.

### 4.2 Differing experiences

However, there were certain differences between the experiences of Mom B, who is Asian, and the experiences of the other parents, who are African American. One example is the use of eye contact; while Parents C and D mentioned encouraging their child to learn eye contact, Mom B explained how she never forced her son to make eye contact. This corresponds with the findings of Tek and Landa (2012), which found that in Asian families, teaching eye contact is not a priority, and children may not learn eye contact. Another difference was found regarding the use of reinforcements. Parents A, C, and D mentioned they reinforced their child’s behavior through prizes, physical gestures, and praise. On the other hand, Mom B noted she does not reinforce her son’s behavior and instead relies on her bond with her child to teach him skills.

Aside from mentions of culture, there were other unique concepts that Mom B provided. She was the only parent who acknowledged in-depth the concepts of Lockwood et al. (2013), who found that autism would impair cognitive perspective-taking but not empathy. While she explained how her child would struggle with perspective-taking, she emphasized her child’s caring side, such as how he would protect his mother and become concerned over how she felt. She also mentioned how she recognized that her child’s developmental milestones differed from typically developing children, consistent with Tek and Landa (2012) regarding the differences in developmental milestones identified in culturally diverse families. Finally, Mom B brought up her faith and the beliefs in the family, with her being Muslim and her husband being Christian. She explained that due to mixed beliefs, she wants her son to develop his own beliefs and have his own understanding of what belief is. The emphasis on religious beliefs corresponds with Luong et al.’s (2009) findings that Asian parents used faith and religion as part of their parenting.

### 4.3 ToMI scores

The parents’ ToMI scores also provided some context to their parenting. Of particular interest were the scores from Parents B, C, and D. With Mom A only completing the first interview and providing short answers, there was not enough context for her ToMI score of 87. In addition, Child A is nonverbal and thus might experience ToM differently from the other verbal children in the study.

Mom B had the second-highest score of 93 out of 130, which was close to the highest score. She provided detailed answers to questions and gave a lot of praise for her son. Her answers were empathetic, and she had a strong understanding of her child’s experiences. Since she brought up her own potential neurodivergence, her connection to her child’s feelings could be explained by the double-empathy hypothesis proposed by Milton (2012), which states neurodivergent people have an easier time taking each other’s perspectives. The other parents, who did not mention having an ADHD or ASD diagnosis, did not provide the same in-depth elaboration regarding their child’s perspective and feelings.

Mom C had the highest score of 96, which corroborated the enthusiastic tone and language of her interview. She would smile and laugh while answering questions, such as when talking about her caregiver job and listing how she would help her child. She gave her child a lot of credit for his development and provided many strategies to encourage his growth.

Meanwhile, Parents D had the lowest ToMI score of 59. Dad D answered the lowest score for most of the questions on the inventory; he also provided short answers to the interview questions with less elaboration than other parents. Mom D also showed less optimism in her answers, focusing more on her child’s deficits compared to the higher emphasis on strengths from the other parents. When asked about her child’s skills at taking turns and using gestures, she responded, “he’s not very good at it,” without further elaboration. She did not provide a verbal answer to the question about her child’s proficiency at reading faces and only shook her head.

While parents did not provide the whole context of their situation, meaning precautions must be taken to prevent faulty assumptions, the parents’ demographic information offers some explanation for their ToMI answers. For example, Parents D have an annual income of $35,000, with Dad D having a high school education level and working as a full-time contractor doing construction work. Dyches et al. (2004) mentioned how racial and disability stigma and living in low-wealth communities could hinder access to healthcare and education, preventing African American children from receiving adequate help from schools or doctors. In addition to the lack of resources and income needed for proper treatment, there is a stigma surrounding disability in African American families, which results in more negative perceptions of children’s ASD (Burkett et al., 2015). On the other hand, Mom B reported having a Ph.D., and Mom C reported an annual income of $103,000, allowing for better access to treatment and therapy.

### 4.4 Limitations and future directions

Other than Mom B, the other parents did not expand upon their experiences from a cultural and ethnic lens. Mom B described how her Kazakh culture shaped how she taught her son developmental skills, but the others did not describe how their culture affected their parenting and their child’s development. Parents also did not explain the role of racial stigma in their experiences, as none of the questions in the interview provided opportunities to expand on this. It would be crucial to examine further how experiences with cultural norms and stigma affect these parental experiences. Future research should include questions more specifically focused on how parents’ culture plays a role in their child’s development and their journey to help their child grow. In addition, considering the role racial stigma plays in hindering African American children’s access to treatment and care, asking questions regarding barriers to receiving care from doctors could allow African American parents to mention race in their experiences.

While some of the parents expressed positive experiences, the questions in the interview focused on the developmental delays of autistic children of color and how parents can help their children develop those skills. Another facet that would be important to examine is how these parents can use their child’s strengths to foster development rather than focusing on weaknesses.

Additionally, the focus of the study on ToM did not consider the other factors that can affect perspective-taking and social skills in autism. When the initial methodology included false-belief tasks, some of the autistic children passed the Sally-Anne and Unexpected Contents tasks. Some of the parents’ answers to the ToMI and the high ToMI scores for Moms B and C also showed their child had developed ToM, contradicting early theories of a ToM deficit in autism (Baron-Cohen et al., 1985). Future studies should include the newer findings on perspective-taking and empathy development in autistic people.

The study only interviewed four families, and one parent did not respond to the second interview. Due to the small sample, the initial scope of the study could not be completed, as four parents would not provide enough data for a regression analysis on predicting children’s ToM ability based on parental perspective-taking teaching strategies. Additionally, the ToMI measure is a quantitative measure typically used for larger samples, and the ToMI is usually coupled with other ToM measures from different sources to provide a complete picture of a child’s ToM ability. Considering the only ToM measure in the study was solely a parental measure, and there were too few participants for full quantitative analysis, the ToMI scores were a closer reflection of parental attitudes towards their child than the child’s actual ToM ability.

Moreover, the study only included Asian and African American parents, and all the children were male and monolingual. Most schools and organizations that were contacted stated they served mostly White children, so reaching out to communities of color would require other methods. These challenges with recruitment would need to be addressed to adequately reach out to communities and increase sample sizes for such studies. An important factor to mention is that due to a history of medical mistreatment and racial stigma from doctors and researchers, there is a mistrust of the medical field among communities of color and a reluctance to participate in studies (Otado et al., 2015). Therefore, future studies must ensure researchers can adequately reach out to underrepresented communities as advocates and work to serve their best interests. Mom B touched upon the subject in her interview:

They’re not going to participate because of the stigma of the study and everything else. They’re just dealing with a lot of things and like big problems right now so they cannot do it… It’s a different type of stigma, it’s such a big stigma and everything else. And it’s such a bunch of stuff about fixing a child and not understanding. They do not want to believe.

Some of the language initially used in the recruitment materials was also called out by other autism researchers, such as the use of the term “high-functioning” and person-first language (child with autism) instead of identity-first language (autistic child). However, the IRB instructed the researchers to use person-first language, suggesting a disconnect between the language used in the research field and the language the community prefers. That said, it is important to recognize that the autistic community is heterogeneous in its perspectives. While many people in the autism community prefer identity-first language, others in the community prefer person-first language (Bury et al., 2020). It is crucial to recognize these differences in views when being advocates to preserve autistic people’s rights to self-determination with the appropriate and inclusive language (Botha et al., 2021).

### 4.5 Conclusion and recommendations

Since research on ToM is done predominantly with White children, it is unknown how ToM exhibits itself in different ethnicities. This is problematic, considering treatment of autism is thought to be one-size-fits-all, but it is not. Because autism research rarely considers the experiences of non-White ethnicities, there is little knowledge of parental experiences raising autistic children in those communities. If further insights on the topic reveal certain strategies outlined in this study would be beneficial, parents can be coached to use those strategies to help their child, similar to the coaching done by Mom B with parents in her community. Thus, when addressing something like ToM, interventions should consider parental experiences influenced by culture.

Knowing the experiences of these parents would allow for their experiences and stories to be shared. The intersection of race/ethnicity and neurodivergence has not gotten much awareness, and those communities have been underrepresented in research. If future work expanding on these stories finds unique experiences within the community, this could lead to developing interventions tailored explicitly to autistic children of color. These interventions could branch off from current autism therapy, such as applied behavior analysis (ABA), a therapy used to treat autism. ABA involves teaching children how to engage in neurotypical, or socially normal, behavior such as behaving socially and developing fine motor skills (Axelrod et al., 2012). The option of different types of therapy is especially relevant considering that while some people in the autism community view ABA as a helpful therapy, others view it as a harmful therapy (McGill and Robinson, 2020). Knowing the cultural differences in parental experiences could help incorporate strategies to teach perspective-taking considering the differences among ethnicities. Considering ABA is heavily based on behavior modeling, teaching children perspective-taking could be another step in figuring out how to improve autism symptoms such as impaired ToM.

## Data availability statement

The raw data supporting the conclusions of this article will be made available by the authors, without undue reservation. To protect confidentiality, the participants’ identifying information (such as names) will be removed from any transcripts and/or videos provided.

## Ethics statement

The studies involving humans were approved by Dr. Sherrill Sellers, Institutional Review Board for Human Subjects Research, Miami University. The studies were conducted in accordance with the local legislation and institutional requirements. Written informed consent for participation in this study was provided by the participants’ legal guardians/next of kin. Written informed consent was obtained from the individual(s), and minor(s)’ legal guardian/next of kin, for the publication of any potentially identifiable images or data included in this article.

## Author contributions

AM: Writing – original draft. YH: Writing – review & editing.

## Supplementary material

Supplementary Table S1 The demographic details of the four families.
