# Supplementary material for: Parental strategies to promote theory of mind development in autistic children of color
Source: Front Psychol. 2024 Apr 18;15:1347504. doi: 10.3389/fpsyg.2024.1347504 (PMC11064013; doi:10.3389/fpsyg.2024.1347504)
Supplement: Supplementary Table S1 — The demographic details of the four families. [file Table_1.pdf]

Supplementary Material

Table 1. The demographic details of the four families

Participant	A	B	C	D
Gender of Parent	Female (Mom A)	Female (Mom B)	Female (Mom C)	Male (Dad D), Female (Mom D)
Gender of Child	Male	Male	Male	Male
Age of Parent	26	36	36	30 (Dad D)
Age of Child	4	5	4	5
Child Diagnosis	Level 3 nonverbal ASD	ASD	Level 1 ASD	ASD
Age of Diagnosis	2.5 to 3 years	3 years	1 year	4 or 5 years
Marital Status	Single	Married	Married	Married
Employment	Employed (working for small business)	Unemployed (caregiver)	Unemployed (caregiver)	Employed full-time (Dad D)
Income Range	$30,000-35,000	Not answered	$103,000	$35,000
Education Level	Some college	Ph.D. (husband has a bachelor's degree)	Bachelor of Science	High school (Dad D)
Race/Ethnicity	African American	Asian/Kazakh (husband is White/native American)	Mixed (Black and White)	Black (Dad D), White (Mom D)
ToMI Score	87	93	96	59
